# Treatment stratification and prognosis assessment using circulating tumor DNA in locally advanced rectal cancer: A systematic review and meta‐analysis

**DOI:** 10.1002/cam4.6434

**Published:** 2023-08-08

**Authors:** Junjie Mi, Rong Wang, Xiaofang Han, Ruijun Ma, Danyu Zhao

**Affiliations:** ^1^ Department of Gastroenterology Shanxi Provincial People's Hospital (The Fifth Hospital of Shanxi Medical University) Taiyuan China; ^2^ Core Laboratory Shanxi Provincial People's Hospital (The Fifth Hospital of Shanxi Medical University) Taiyuan China

**Keywords:** circulating tumor DNA, locally advanced rectal cancer, pathological complete response, prognosis, recurrence

## Abstract

**Background:**

Circulating tumor DNA (ctDNA) is an emerging biomarker for locally advanced rectal cancer (LARC), giving hope for stratified treatment. As the completed studies have small sample sizes and different experimental methods, systematic review and meta‐analysis were performed to explore their role in predicting pathological complete response (pCR), tumor recurrence, and prognosis.

**Methods:**

PubMed, Embase, and the Web of Science were searched for potentially eligible studies published up to September 6, 2022. Pooled relative risk (RR) was calculated to predict pCR and tumor recurrence, and pooled hazard ratio (HR) was calculated to evaluate the prognosis of overall survival (OS), recurrence‐free survival (RFS), and metastasis‐free survival (MRS).

**Results:**

Twelve studies published between 2018 and 2022 included 931 patients, and 2544 serum samples were eventually included in the meta‐analysis. The pooled revealed that ctDNA‐negative patients were more likely to have a pCR (RR = 1.64, 95% confidence interval [CI]: 1.26–2.12). The pooled revealed that ctDNA‐positive patients were at high risk of recurrence (RR = 3.37, 95% CI: 2.34–4.85) and had a poorer prognosis for OS (HR = 3.03, 95% CI: 1.86–4.95), RFS (HR = 7.08, 95% CI: 4.12–12.14), and MRS (HR = 2.77, 95% CI: 2.01–3.83).

**Conclusion:**

ctDNA may be useful for stratifying treatment and assessing prognosis in patients with LARC, but its clinical application still needs to be confirmed in a prospective multicenter study with large samples.

## 1 INTRODUCTION

Colorectal cancer is the third most common cancer in the world with the fourth highest mortality rate, of which rectal cancer accounts for approximately 28%.^1^, ^2^ Neoadjuvant chemoradiotherapy (nCRT) followed by total mesorectal excision (TME) and adjuvant chemotherapy (ACT) is one of the recommended treatment regimens for locally advanced rectal cancer (LARC).^3^, ^4^ About 15%–44% of patients undergoing nCRT will have a pathological complete response (pCR), which means that the tumor is not found in the final surgical specimen, and these patients may have a lower rate of local recurrence and distant metastases and have better overall survival.^5^, ^6^, ^7^, ^8^ The nonoperative management (NOM) has been proposed for patients with pCR, which can improve quality of life.^9^, ^10^, ^11^ Currently, clinical complete response (cCR) is defined as the inability to detect tumors by including magnetic resonance imaging (MRI), endoscopy, and digital rectal examination to guide the selection of patients suitable for NOM.^12^, ^13^ However, the agreement between cCR and pCR is not satisfactory, so there is a clinical need for a biomarker that can accurately predict pCR.^14^

Rectal cancer has a high recurrence rate within 5 years after radical surgery, with Stages II and III recurrence rates of 30% and 50%, respectively.^15^ Early detection of tumor recurrence can facilitate timely treatment and thus improve the patient's prognosis, but the methods currently used in clinical practice to detect tumor recurrence have low sensitivity.^16^, ^17^ MRI is commonly used to diagnose LARC and monitor tumor recurrence with a sensitivity of only 68.3% for local recurrence in LARC patients after nCRT.^18^ In addition, carcinoembryonic antigen (CEA) is commonly used clinically to monitor the recurrence of colorectal cancer, but its sensitivity and specificity are still unsatisfactory.^19^, ^20^ In conclusion, there is still a need to explore new biomarkers for the prediction of LARC recurrence.

Circulation tumor DNA (ctDNA) is the release of apoptotic or necrotic tumors into the bloodstream with genetic variation, and there is a high degree of concordance between the genomic alterations found in tumor tissue.^21^, ^22^ With easy sampling, noninvasive, real‐time, and multiple executions, ctDNA has great potential to guide treatment selection, tumor recurrence monitoring, and prognostic monitoring.^23^, ^24^ Positive ctDNA after radical surgery for curative purposes in esophageal adenocarcinomas and non‐small cell lung cancer carries a high risk of recurrence.^25^, ^26^ In addition, ctDNA‐guided categorical therapy for colon cancer has reduced adjuvant chemotherapy.^27^

A number of studies have explored the role of ctDNA in predicting pCR, tumor recurrence risk, and prognosis in LARC. However, the evidence is still insufficient for direct clinical application due to the small sample size and the different experimental approaches.^28^, ^29^, ^30^, ^31^, ^32^, ^33^, ^34^, ^35^, ^36^, ^37^, ^38^, ^39^ In addition, published studies on the role of ctDNA in LARC have shown inconsistent results. The aim of this study was to systematically review and meta‐analyse the role of ctDNA in predicting pCR, risk of recurrence, and prognosis in patients with LARC.

## 2 MATERIALS AND METHODS

### 2.1 Literature search

This meta‐analysis was carried out according to preferred reporting items for systematic reviews and meta‐analyses guidelines.^40^ This meta‐analysis was registered at the International Prospective Register of Systematic Reviews with the registration number CRD42022367608. We searched PubMed, Embase, and Web of Science for studies on ctDNA predicting pCR, risk of recurrence, and prognosis in LARC patients with a search deadline of September 6, 2022. In addition, the investigators searched for references in the included studies to prevent omissions. Inclusion criteria were as follows: (a) patients were diagnosed with LARC; (b) investigation of the role of ctDNA in the prediction of pCR, tumor recurrence risk, and prognosis; and (c) studies that provided enough information to calculate relative risk (RR) for predicting pCR, tumor recurrence, and hazard ratio (HR) for prognosis. The exclusion criteria were as follows: (a) conference abstracts, reviews, letters, case reports, and nonclinical studies; (b) studies for which relevant raw data were not available; (c) studies involving comorbidities with other malignant diseases; (d) studies involving colon cancer; and (e) studies detecting cell‐free DNA. The study search strategy used a combination of subject terms and free words, and the search strategy was as follows: #1: Circulating Tumor DNA OR ctDNA OR (Circulating Tumor AND DNA) OR (Tumor DNA AND Circulating) OR Cell Free Tumor DNA OR (Cell‐Free Tumor AND DNA) OR (Tumor DNA AND Cell‐Free); #2: Locally advanced rectal cancer OR Rectal cancer OR Rectal Neoplasms; #3: Pathological complete response OR Chemoradiotherapy OR Neoadjuvant chemoradiotherapy OR Prognosis OR Minimal residual disease OR Recurrence risk; #4: #1 and #2 and #3. The Supporting Information contained detailed search process.

### 2.2 Data extraction

Two investigators (J.M., R.W.) independently collected data for each included study by developing a data extraction form, and if the comparison of the extracted data did not agree, the disagreement was resolved by consulting a third person (X.H.). The data extract included basic information about the included studies as follows: first author, year of publication, country, patient count, average age, gender, number of serum samples, sample type, testing methods, number of genes, number of centers, study design, TNM staging, clinical stage, median follow‐up, ctDNA testing time, definition of pCR, long‐term endpoint definition, and treatment strategy. For pCR and the risk of tumor recurrence, the number of ctDNA‐positive patients and the number of ctDNA‐negative patients were extracted. Regarding the prognostic indicators overall survival (OS), recurrence‐free survival (RFS), and metastasis‐free survival (MRS), HR as well as 95% confidence intervals (95% CIs) were extracted for each study. If the relevant data are not available from the original study, we will contact the corresponding author of the original study to obtain the raw data. We used the Newcastle–Ottawa scale to rate the included studies from 0 to 9.

### 2.3 Statistical analysis

The effect sizes for pCR and risk of tumor recurrence were assessed by the Mantel–Haenszel method as RR and 95% CI, and for prognosis as HR and their 95% CI for OS, progression‐free survival (PFS), and MRS. A chi‐squared *Q* test and *I* ^2^ coefficients were also used to quantify the degree of heterogeneity between included studies, with *p* < 0.05 considered a statistically significant difference using a random effects model; otherwise, a fixed effects model was used. Subgroup analysis was performed to investigate the utility of ctDNA testing at various time points in predicting pCR, tumor recurrence risk, and prognosis, as well as to investigate the sources of heterogeneity in this study. There were four subgroups based on the time point of ctDNA testing: baseline, on‐chemoradiotherapy (on‐CRT), presurgery, and postsurgery groups. In addition, we performed sensitivity analyses to test the stability of the meta‐analysis results, and finally, the Egger test and harbor test were used to assess whether there was publication bias in this study. The overall effect test was statistically significant at *p* < 0.05, and all analyses were completed using STATA 17.0 software.

## 3 RESULTS

### 3.1 Characteristics of eligible studies and quality assessment

An initial selection of 549 articles was achieved after careful searching of the databases, as detailed in Figure S1. A total of 931 patients and 2544 serum samples were included in the meta‐analysis of 12 studies published between 2018 and 2022, in which the study characteristics and demographics are summarized in Table 1. The timing of ctDNA testing, the definition of pCR, the definition of long‐term endpoints, and treatment strategies were summarized in Table S1. The results of the assessment of the quality of the included studies by means of the Newcastle–Ottawa scale are shown in Table S2. In addition, the Egger test and Harbord test were used to assess publication bias, and the results showed no publication bias, and sensitivity analysis showed that none of the studies could significantly affect the pooled effect (see Figures S2–S7 for details).

**TABLE 1:** Characteristics of the included studies in this meta‐analysis.

First author	Year of publication	Country	No. of patients	Mean age (range/IQR)	Female (%)	No. of serum samples	Sample type
Sclafani	2018	United Kingdom	59	NA	38.9	59	Plasma
Tie	2019	Australia	159	62	33	462	Plasma
Appelt	2020	United Kingdom	146	64 (57–69)	36	146	Serum
Khakoo	2020	United Kingdom	47	59 (30–83)	39	243	Plasma
Murahashi	2020	Japan	85	60 (52–69)	23.5	222	Plasma
Pazdirek	2020	Czechia	36	64.1 (30–83)	25	NA	Plasma
Vidal	2021	Spain	62	60 (33–75)	35.5	97	Plasma
Zhou	2021	China	104	60 (26–74)	35.6	384	Plasma
Wang	2021	China	119	57	15	531	Plasma
McDuff	2021	USA	29	54 (45–78)	48	47	Plasma
Roesel	2022	Switzerland	25	65 (59–70)	12	157	Plasma
Liu	2022	China	60	53 (31–67)	30	196	Plasma

Testing methods	No. of genes	No. of centers	Study design	Clinical stage	TNM staging	Median follow‐up (range/IQR, months)
dd PCR	2	Multicenter	Retrospectively	NA	NA	NA
NGS	15	Multicenter	Prospectively	II, III	cT3‐4N0 or cTanyN1‐2	24 (1–55)
dd PCR	1	Multicenter	Retrospectively	NA	T3‐4 N0‐2 M0	127.2 (110.4–138)
dd PCR	6	Single center	Prospectively	I–III	cT3‐4 and/or N+	26.4
NGS	14	Single center	Prospectively	II, III	cT3‐4N0 or cTanyN+	NA
BEAMing	6	Single center	Prospectively	II, III	T1‐4/N0‐2	36
NGS	NA	Multicentric	Prospectively	NA	ypT0‐4/ypN0‐2	38 (2.3–51.5)
NGS	733	Multicenter	Prospectively	II, III	cT4Nany/cTanyN1b‐2	18.8 (3.1–21.3)
NGS	422	Single center	Prospectively	IIA–IIIC	cT3‐4/N0‐2/M0	21.5 (1.2–30.8)
dd PCR	NA	Multicenter	Retrospectively	II, III	ypT0‐4/N0‐2b/M0‐1b	20 (14–43)
NGS	14	Multicenter	Prospectively	NA	ypT0‐4/ypN0‐2	14 (8–14)
NGS	22	Multicenter	Prospectively	II, III	cT3‐4N0/cTanyN1‐2	33.3 (9.6–42.4)

Abbreviations: BEAMing, bead emulsion amplification magnetic; dd PCR, droplet digital polymerase chain reaction; IQR, interquartile range; NA, not available; NGS, next‐generation sequencing; PCR, polymerase chain reaction.

### 3.2 ctDNA prediction of pCR for LARC

**FIGURE 1:**
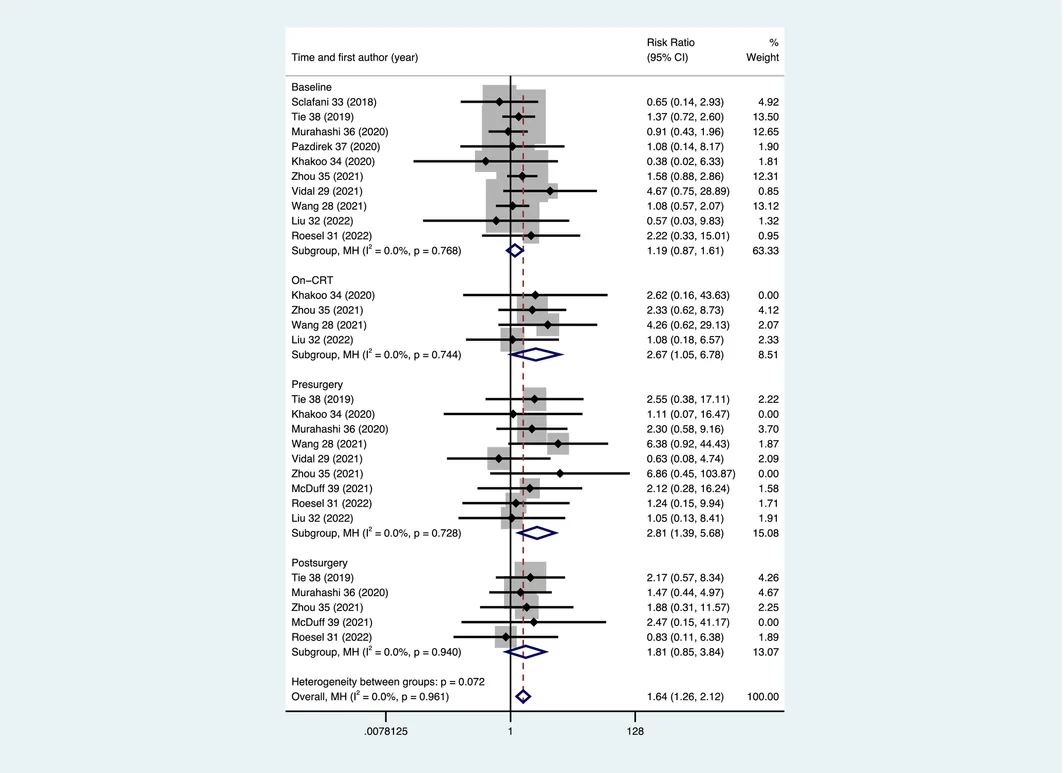
Forest plot of pooled effect for relative risk in circulating tumor DNA prediction of pathological complete response.

A total of 11 studies were pooled to analyze ctDNA prediction of pCR in Figure 1.^28^, ^29^, ^31^, ^32^, ^33^, ^34^, ^35^, ^36^, ^37^, ^38^, ^39^ ctDNA‐negative patients were more likely to have a pCR during LARC treatment (RR = 1.64 95% CI: 1.26–2.12, *p* < 0.05). In the subgroup analysis, baseline included 10 studies, and ctDNA did not predict pCR (RR = 1.19, 95% CI: 0.87–1.61, *p* > 0.05).^28^, ^29^, ^31^, ^32^, ^33^, ^34^, ^35^, ^36^, ^37^, ^38^ On‐CRT included four studies, and ctDNA‐negative patients were more prone to pCR (RR = 2.67, 95% CI: 1.05–6.78, *p* < 0.05).^28^, ^32^, ^34^, ^35^ Presurgery included nine studies, and ctDNA‐negative patients were more prone to pCR (RR = 2.81, 95% CI: 1.39–5.68, *p* < 0.05).^28^, ^29^, ^31^, ^32^, ^34^, ^35^, ^36^, ^38^, ^39^ Postsurgery included five studies, and ctDNA did not predict pCR (RR = 1.81, 95% CI: 0.85–3.84).^31^, ^35^, ^36^, ^38^, ^39^

### 3.3 ctDNA predicting the risk of LARC recurrence

**FIGURE 2:**
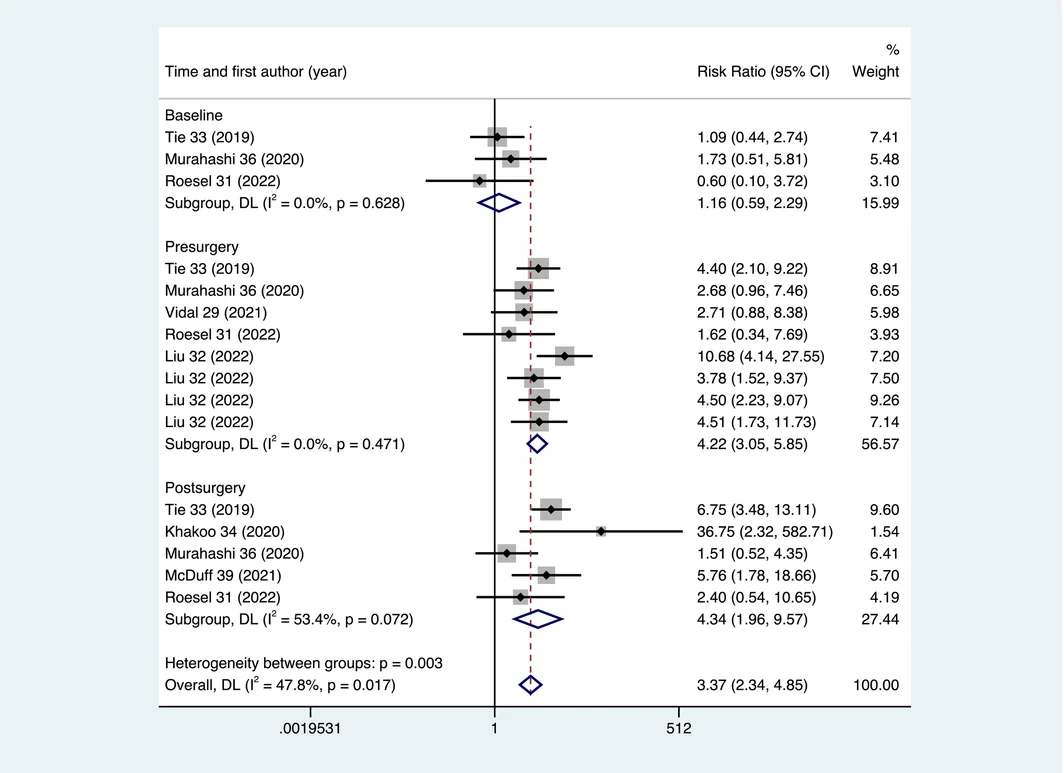
Forest plot of pooled effect for relative risk in circulating tumor DNA prediction of locally advanced rectal cancer recurrence.

A total of seven studies were included in the pooled analysis of ctDNA to predict the risk of LARC recurrence, of which Liu's study used different methods to detect ctDNA and each method was included in the meta‐analysis to assess LARC recurrence at presurgery.^29^, ^31^, ^32^, ^34^, ^36^, ^38^, ^39^ ctDNA‐positive patients had a higher risk of LARC recurrence in Figure 2 (RR = 3.37, 95% CI: 2.34–4.85). Baseline included three studies, and ctDNA did not predict LARC recurrence (RR = 1.16, 95% CI: 0.59–2.29, *p* > 0.05).^31^, ^36^, ^38^ Presurgery included five studies with ctDNA‐positive patients at a higher risk of LARC recurrence (RR = 4.22, 95% CI: 3.05–5.85, *p* < 0.05).^29^, ^31^, ^32^, ^36^, ^38^ Postsurgery included five studies with ctDNA‐positive patients at a higher risk of LARC recurrence (RR = 4.34, 95%CI: 1.96–9.57, *p* < 0.05).^31^, ^34^, ^36^, ^38^, ^39^

### 3.4 Prediction of OS by ctDNA

**FIGURE 3:**
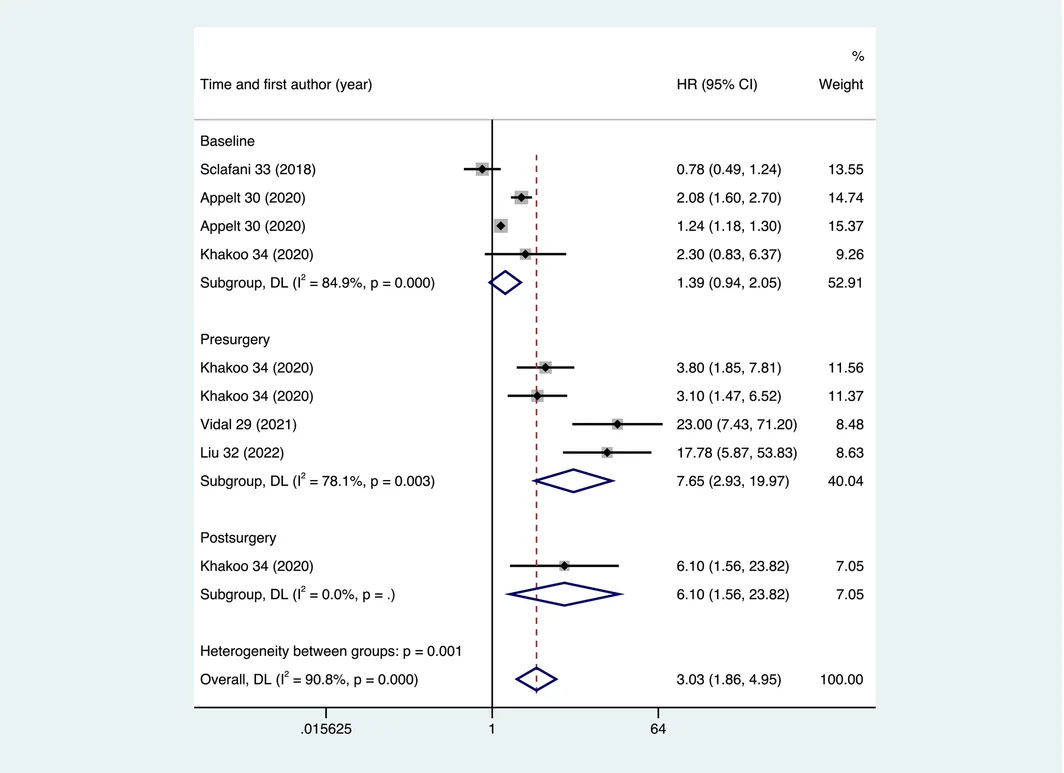
Forest plot on circulating tumor DNA prediction of overall survival with a pooled effect hazard ratio.

For prognosis, five studies were pooled to analyze ctDNA predicting OS.^29^, ^30^, ^32^, ^33^, ^34^ The Appelt study used hypermethylated ctDNA and hypermethylation quantitative load methods to assess OS, respectively, both of which were included separately in the pooled analysis, and in the Khakoo study predicted OS at presurgery including mid‐CRT and completion of CRT, respectively.^30^, ^34^ ctDNA‐positive patients had a poorer prognosis for OS as shown in Figure 3 (HR = 3.03, 95% CI:1.86–4.95, *p* < 0.05). In subgroup analysis, ctDNA detection at baseline did not predict OS (HR = 1.39, 95% CI: 0.94–2.05, *p* > 0.05).^30^, ^33^, ^34^ At presurgery, ctDNA positivity had poor OS (HR = 7.65, 95% CI: 2.93–19.97, *p* < 0.05) and at postsurgery, only one study showed that ctDNA positivity had poor OS (HR = 6.10, 95% CI: 1.56–23.82).^29^, ^32^, ^34^

### 3.5 Prediction of RFS by ctDNA

**FIGURE 4:**
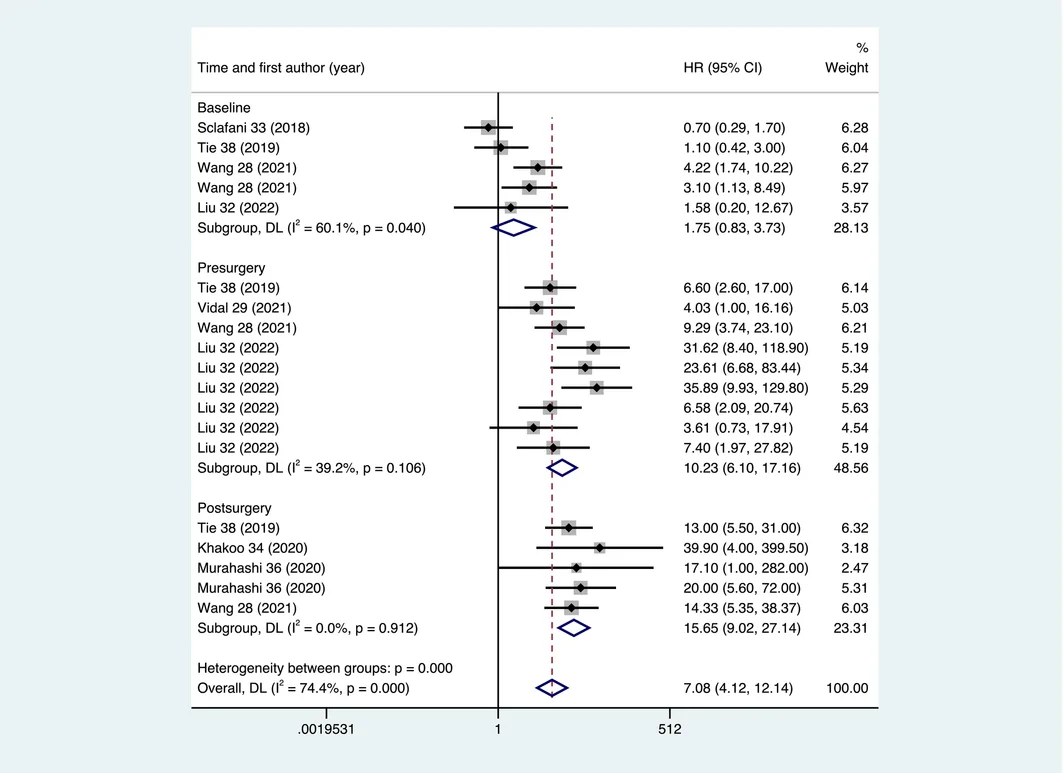
Forest plot on circulating tumor DNA prediction of progression‐free survival with a pooled effect hazard ratio.

Seven studies were pooled to analyze ctDNA as a prediction of RFS, with Liu, Wang, and Murahashi's study using a number of different methods to detect ctDNA as a predictor of RFS, each of which was included separately in the pooled analysis.^28^, ^29^, ^32^, ^33^, ^34^, ^36^, ^38^ For the estimation of RFS, the results of disease‐free survival and PFS were included. ctDNA‐positive patients had a poorer RFS in Figure 4 (HR = 7.08, 95% CI: 4.12–12.14, *p* < 0.05). ctDNA‐positive patients had poorer RFS at presurgery and postsurgery (HR = 10.23 95% CI: 6.10–17.16, *p* < 0.05; HR = 15.65 95% CI: 9.02–27.14, *p* < 0.05). For multivariate analysis, ctDNA‐positive patients had a poorer prognosis for RFS as detailed in Figure S8 (HR = 9.66, 95% CI: 5.31–17.59, *p* < 0.05).^28^, ^32^, ^36^, ^38^

### 3.6 Prediction of MRS by ctDNA

A total of four studies were pooled to analyze the prognosis of ctDNA predicting MRS, and ctDNA‐positive patients had a poorer prognosis for MRS in Figure S9 (HR = 2.77, 95% CI: 2.01–3.83, *p* < 0.05).^30^, ^32^, ^34^, ^35^ A total of three studies were pooled for analysis at the time of baseline for MRS predicted by ctDNA, with Appelt and Zhou's study using different methods for setting ctDNA positivity thresholds, each of which was included separately in the pooled analysis.^30^, ^34^, ^35^ At baseline, ctDNA‐positive patients had a poorer prognosis for MRS (HR = 1.31, 95% CI: 1.10–1.56, *p* < 0.05). Two and three studies were pooled for analysis at on‐CRT and presurgery, respectively, with Zhou's study using different methods for setting ctDNA positivity thresholds and Khakoo's study including different time points for detecting ctDNA, each of which was included separately in the pooled analysis.^32^, ^34^, ^35^ Patients who had positive ctDNA during CRT and before surgery had a poor prognosis for MRS (HR = 3.66, 95%CI:1.80–7.45, *p* < 0.05; HR = 11.53, 95% CI: 5.97–22.29, *p* < 0.05). Only Zhou's study evaluated ctDNA predictive MRS at postsurgery, where ctDNA positivity thresholds were set using different methods, each of which was included separately in the pooled analysis.^35^ Patients with positive ctDNA at postsurgery had a poorer prognosis for MRS (HR = 24.73, 95% CI: 2.49–245.75, *p* < 0.05).

## 4 DISCUSSION

The standards of treatment recommended by guidelines for patients with LARC are TME and ACT after nCRT, and although most patients benefit from treatment as recommended by guidelines, each patient's tumor is highly heterogeneous and responds to treatment in a highly different way.^41^, ^42^ Patients who do not respond well to treatment not only do not benefit from standard treatment but also suffer from the toxic side effects of chemoradiotherapy, and the damage to normal body functions caused by surgery.^43^ ctDNA has been shown to be used to guide patient treatment selection, detect tumor recurrence, and predict prognosis, bringing hope for individualized and precise treatment of tumor patients.^44^, ^45^ Our meta‐analysis demonstrated that ctDNA‐negative patients are more prone to pCR, have a lower risk of tumor recurrence, have a better prognosis, and may be considered for NOM as a treatment option in this group of patients. Patients with positive ctDNA had a higher risk of tumor recurrence, and such patients may require intensive follow‐up to receive timely treatment. ctDNA could also be used to predict LARC prognosis, with ctDNA‐positive patients having a worse OS, a worse RFS, and a worse MRS.

Although TME is recommended by guidelines for the treatment of LARC, it can cause bowel, sexual, and genitourinary dysfunctions that can seriously affect the quality of life of patients.^46^, ^47^ In addition, a 14‐year multicenter randomized study compared the long‐term effects of TME and TME plus radiotherapy on bowel function by low anterior resection syndrome score (LARS score), with major LARS indicating severe bowel dysfunction. Major LARS was reported by 46% of all patients (56% radiotherapy plus TME vs. 35% TME), and radiotherapy plus TME (odds ratio = 3.0; CI, 1.3–6.9) increased the risk of major LARS.^46^ NOM can avoid the complications of surgery for a high quality of life and has become one of the treatment options for LARC patients.^48^, ^49^, ^50^, ^51^ Patients with LARC presented a variable response to treatment after nCRT, with 24.4% presenting with pCR, approximately 45% with tumor downstaging and the remaining patients presenting with no or partial response.^8^, ^52^ The critical aspect of selecting NOM is the correct identification of those patients who will develop pCR after nCRT. This meta‐analysis showed that ctDNA‐negative patients were more prone to developing pCR at on‐CRT and presurgery.

Accurate assessment of the risk of relapse after LARC treatment can prevent over‐treatment of patients at low risk of relapse and undertreatment of patients at high risk of relapse. In addition, the high rate of metastatic recurrence in patients after LARC surgery is an important reason for their high mortality rate. It is therefore clinically important to find a biomarker that can detect tumor recurrence in a timely manner with high accuracy and simplicity. Colonoscopy and CEA are routinely used to assess recurrence after LARC treatment but have shortcomings in clinical practice. Colonoscopy and biopsy, as routine methods for colorectal cancer diagnosis, have high sensitivity and specificity, but some patients resist colonoscopy due to fear of colonoscopy, and complications.^53^, ^54^, ^55^ CEA is the only biomarker recommended by the National Comprehensive Cancer Network for postoperative surveillance of colorectal cancer recurrence, but its sensitivity and specificity are not satisfactory.^56^, ^57^, ^58^ ctDNA provides information on tumor‐specific genetic alterations, including single‐nucleotide site variants, copy number alterations, gene fusions, and epigenetic alterations, and is noninvasive and easy to use multiple times, making it an ideal biomarker for monitoring tumor recurrence. Positive ctDNA has been shown to be associated with a high risk of recurrence after colorectal cancer surgery.^59^, ^60^, ^61^ In addition, the Liu study showed that the area under the receiver operating characteristic curve for ctDNA predicting LARC recurrence was 0.87 higher than the CEA (0.52) and CA199 (0.49).^32^ The pooled analysis of this study showed a higher risk of tumor recurrence for ctDNA positivity at presurgery, suggesting that this group of patients may respond poorly to preoperative nCRT treatment and that surgical and postoperative treatment should be intensified in this group of patients. At postsurgery, ctDNA‐positive patients also had a higher risk of tumor recurrence, suggesting that these patients may require boosted postoperative ACT and frequent follow‐ups. Unfortunately, ctDNA did not predict tumor recurrence at baseline.

ctDNA was a good predictor of OS, and patients who tested positive for ctDNA had a worse prognosis for OS. However, ctDNA testing at different time points showed different results in predicting OS, and ctDNA testing at baseline was not associated with OS. Positive ctDNA at presurgery had a poor OS, so this group of patients may not benefit from standard treatment and may require intensive treatment. RFS reflects tumor progression, which is largely responsible for tumor‐related deaths, and the need to change treatment regimens after tumor progression, making ultimate survival dependent on subsequent treatment regimens. Our pooled study demonstrated that ctDNA‐positive patients had worse RFS. In addition, presurgical ctDNA and postsurgical ctDNA detected recurrence 10.2 and 2.6 months ahead of imaging, respectively.^32^, ^34^ In the subgroup analysis, ctDNA‐positive patients had poorer RFS at presurgery and postsurgery. Patients who were ctDNA‐positive at presurgery and postsurgery were more prone to developing progression, so these patients need to be monitored more closely and may require a change of treatment regimen after progression. Metastatic recurrence in patients after LARC surgery is 25% and is a major contributor to their mortality, making MFS an important indicator to assess the prognostic survival of LARC patients.^62^, ^63^ This pooled study demonstrated that ctDNA‐positive patients had poorer MRS. In subgroup analyses at baseline, on‐CRT, presurgery, and postsurgery, ctDNA‐positive patients all had poorer MRS, so that positive ctDNA patients were more likely to have metastases regardless of when ctDNA was tested in LARC treatment. In addition, the association between positive ctDNA and distant tumor metastases strengthened as the time point for ctDNA testing moved forward. In conclusion, ctDNA was a good indicator to assess the prognosis of LARC and stronger treatment and closer follow‐up were needed for ctDNA‐positive patients.

Although first meta‐analysis demonstrated the potential usefulness of ctDNA in predicting pCR, tumor recurrence, and prognosis in LARC, the following shortcomings exist. First, the number of studies and sample size included in this meta‐analysis as well as in the subgroup analysis was relatively small. Second, there was heterogeneity in the meta‐analysis as well as in the subgroup analysis. Third, most of the studies included in the assessment of prognosis had short follow‐up periods, and in particular, the follow‐up period for assessing OS was not long enough.

In conclusion, this meta‐analysis demonstrated that ctDNA‐negative patients are more prone to pCR, have a lower risk of tumor recurrence, and may be considered for NOM as a treatment option. This study also showed a higher risk of tumor recurrence in ctDNA‐positive patients at presurgery and postsurgery, suggesting that these patients may respond poorly to treatment and should be closely followed. ctDNA could also be used to predict LARC prognosis, with ctDNA‐positive patients having a worse OS, a worse RFS, and a worse MRS. The sample size of known studies is small, and large‐scale multicenter prospective studies are still needed to determine the role and clinical application of ctDNA in LARC.

## AUTHOR CONTRIBUTIONS

**Junjie Mi:** Conceptualization (lead); supervision (lead); writing – original draft (lead). **Rong Wang:** Data curation (equal); formal analysis (equal); software (equal); writing – original draft (supporting). **Xiaofang Han:** Investigation (equal); writing – original draft (equal). **Ruijun Ma:** Formal analysis (equal); software (equal). **Danyu Zhao:** Formal analysis (equal); investigation (equal); software (equal).

## CONFLICT OF INTEREST STATEMENT

There are no conflicts of interest.

## Supporting information


Data S1.

Data S2.

## Data Availability

All data from this study are available through the corresponding author.
