# Supplementary material for: Treatment stratification and prognosis assessment using circulating tumor DNA in locally advanced rectal cancer: A systematic review and meta‐analysis
Source: Cancer Med. 2023 Aug 8;12(17):17934–44. doi: 10.1002/cam4.6434 (PMC10523996; doi:10.1002/cam4.6434)
Supplement: Supplementary file 1 — Data S1. [file CAM4-12-17934-s001.docx]

**Supplementary Data**


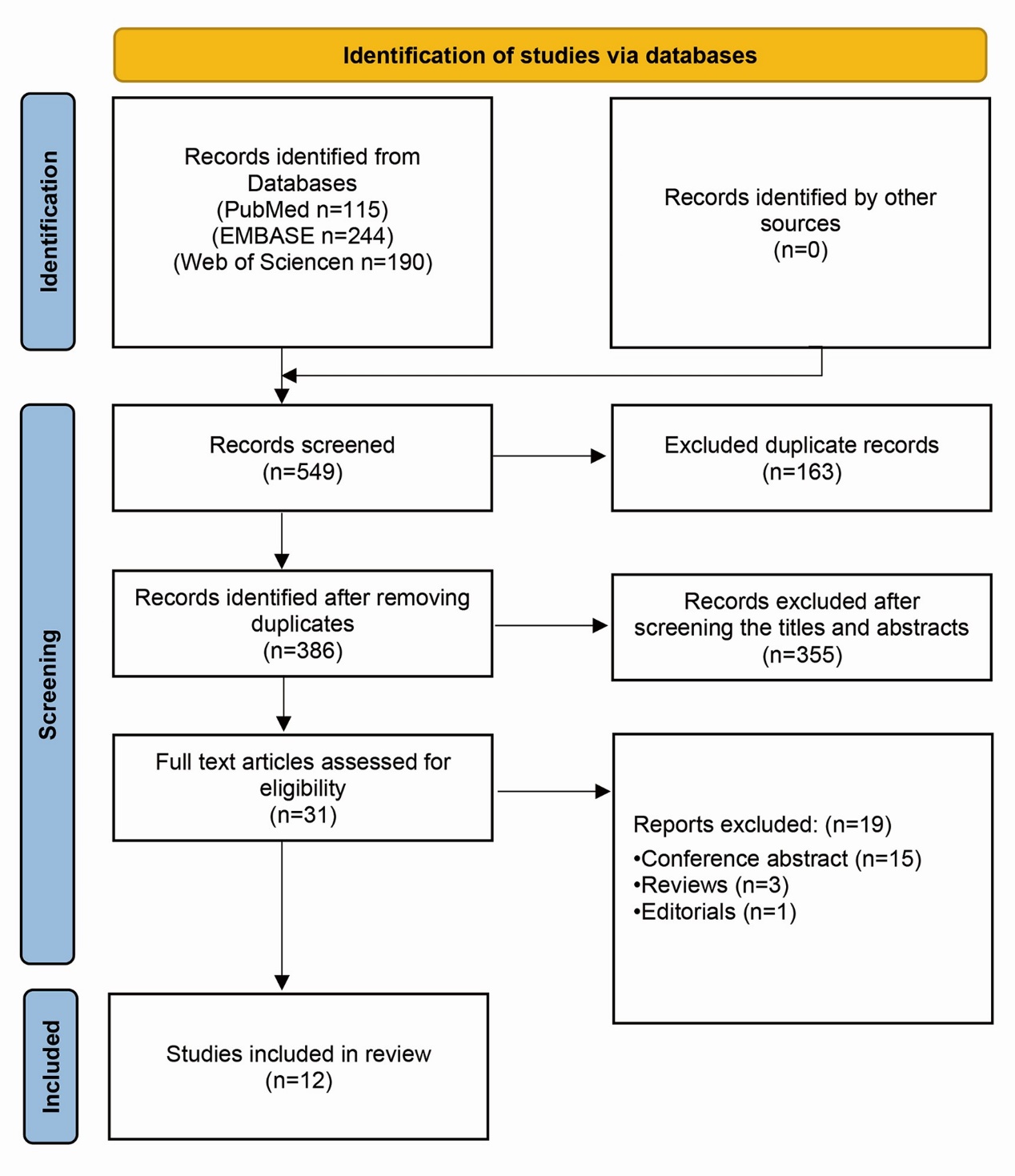


**Supplementary figure 1.** Literature search and study selection flow diagram for systematic reviews


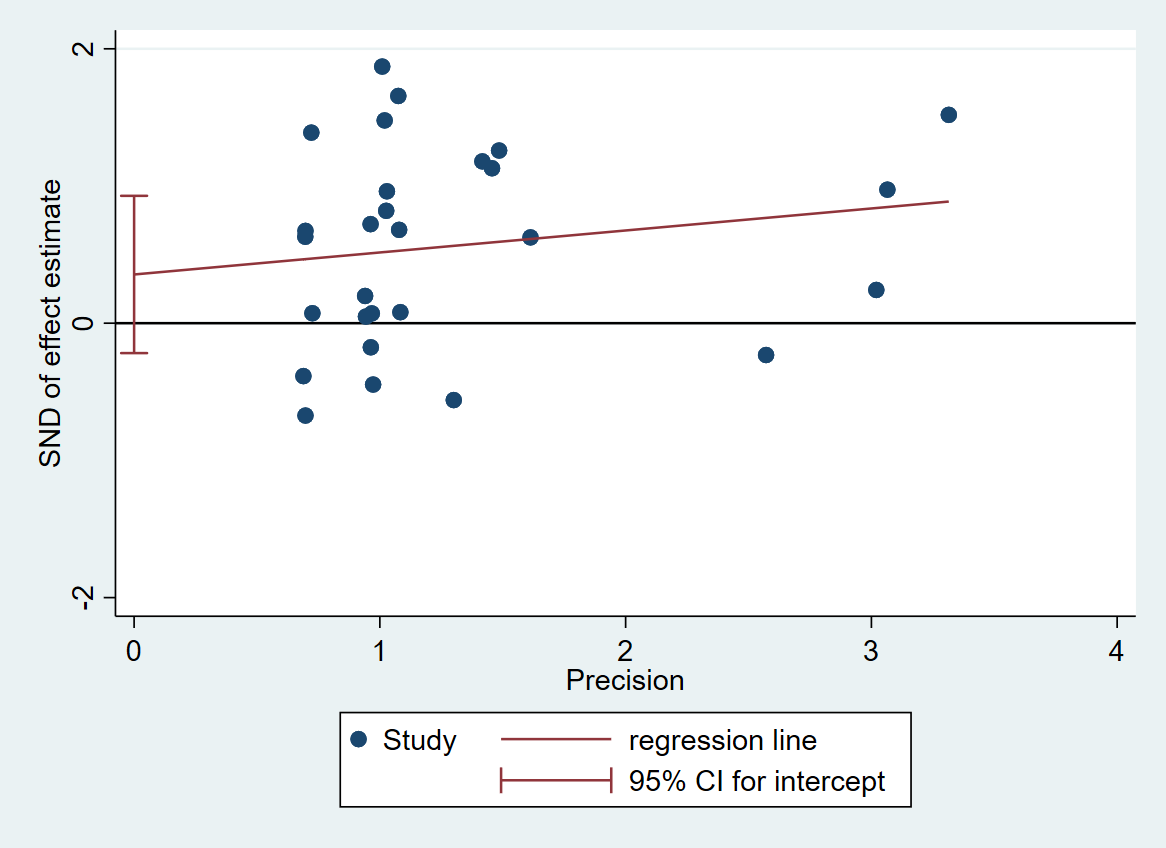


**Supplementary figure 2.** The Egger test for ctDNA prediction of pathological complete response.


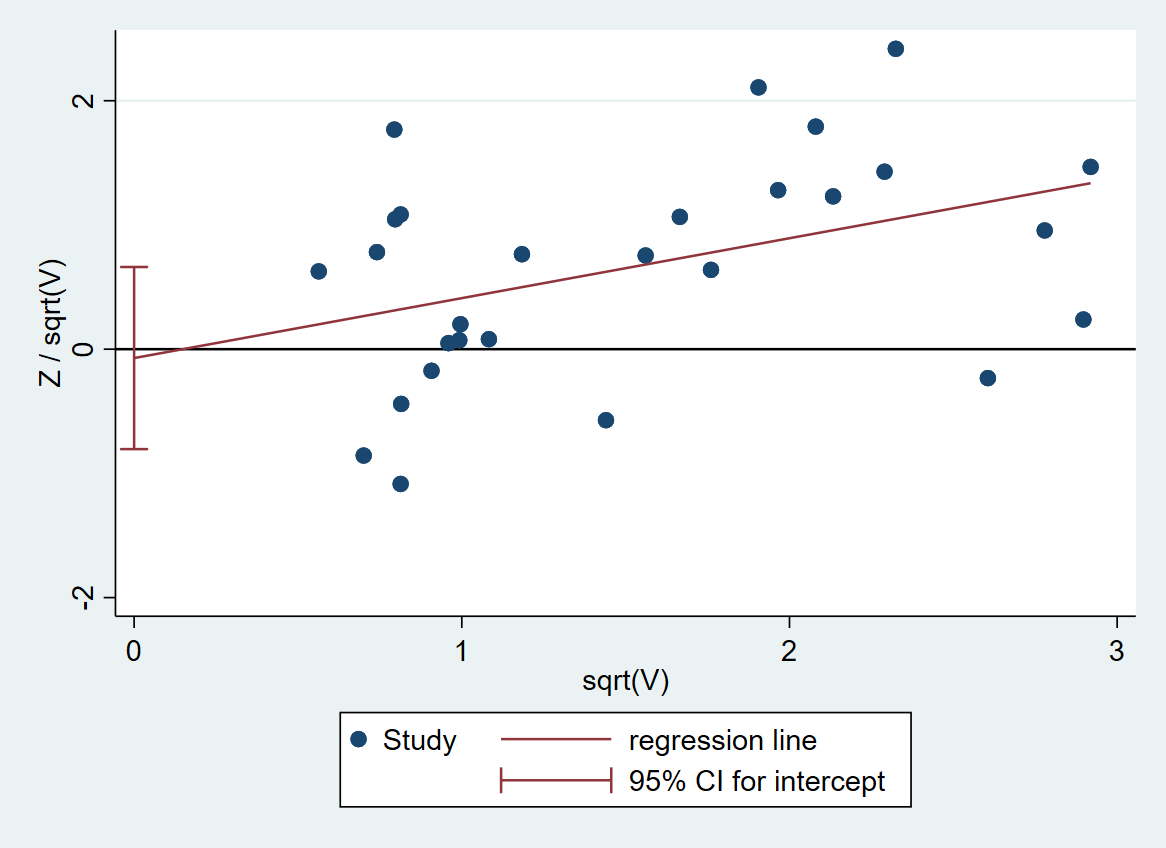


**Supplementary figure 3.** The Harbord test for ctDNA prediction of pathological complete response.


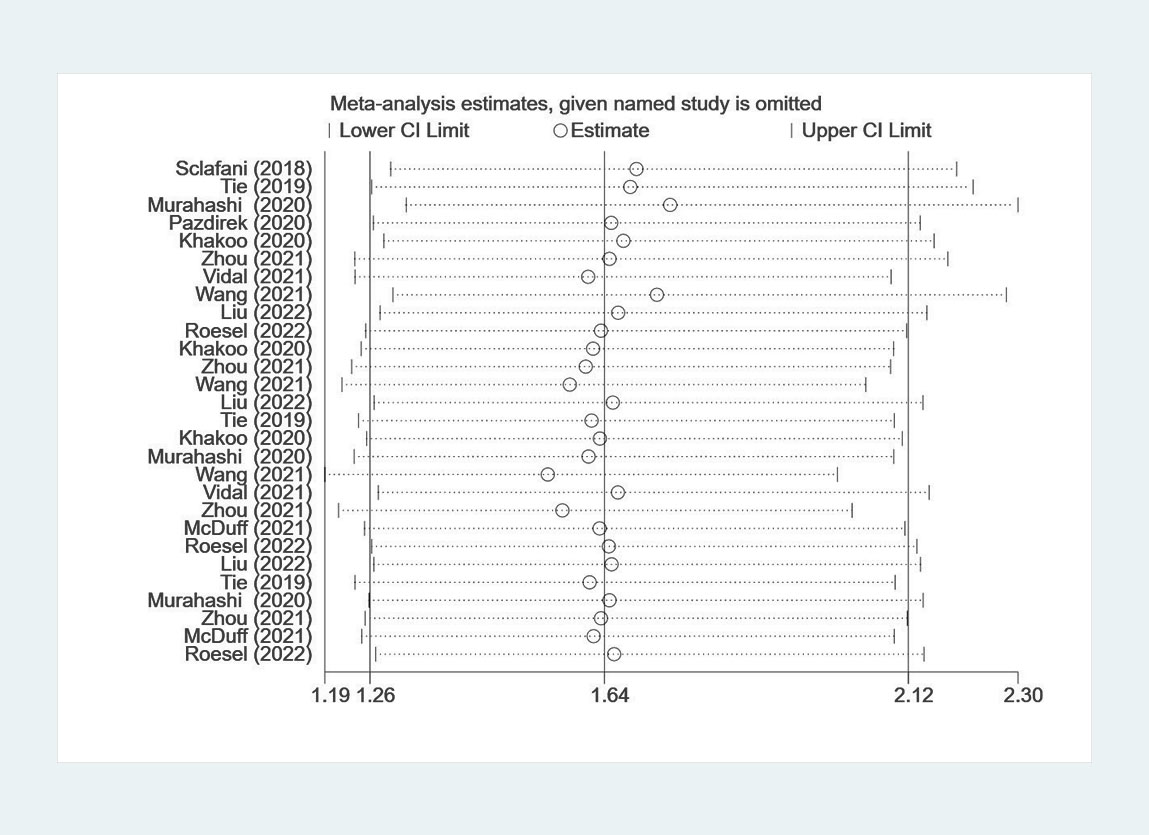


**Supplementary figure 4.** The sensitivity analysis for ctDNA prediction of pathological complete response.


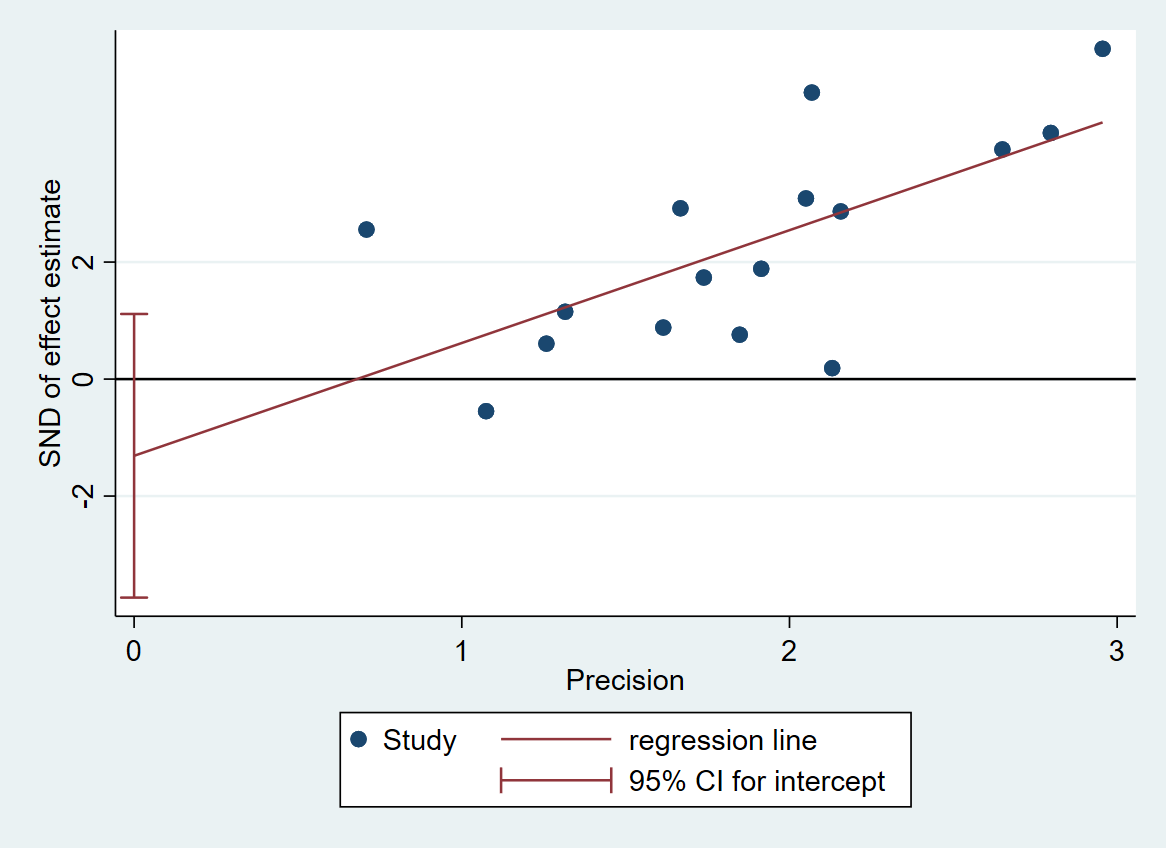


**Supplementary figure 5.** The Egger test for ctDNA prediction of locally advanced rectal cancer recurrence.


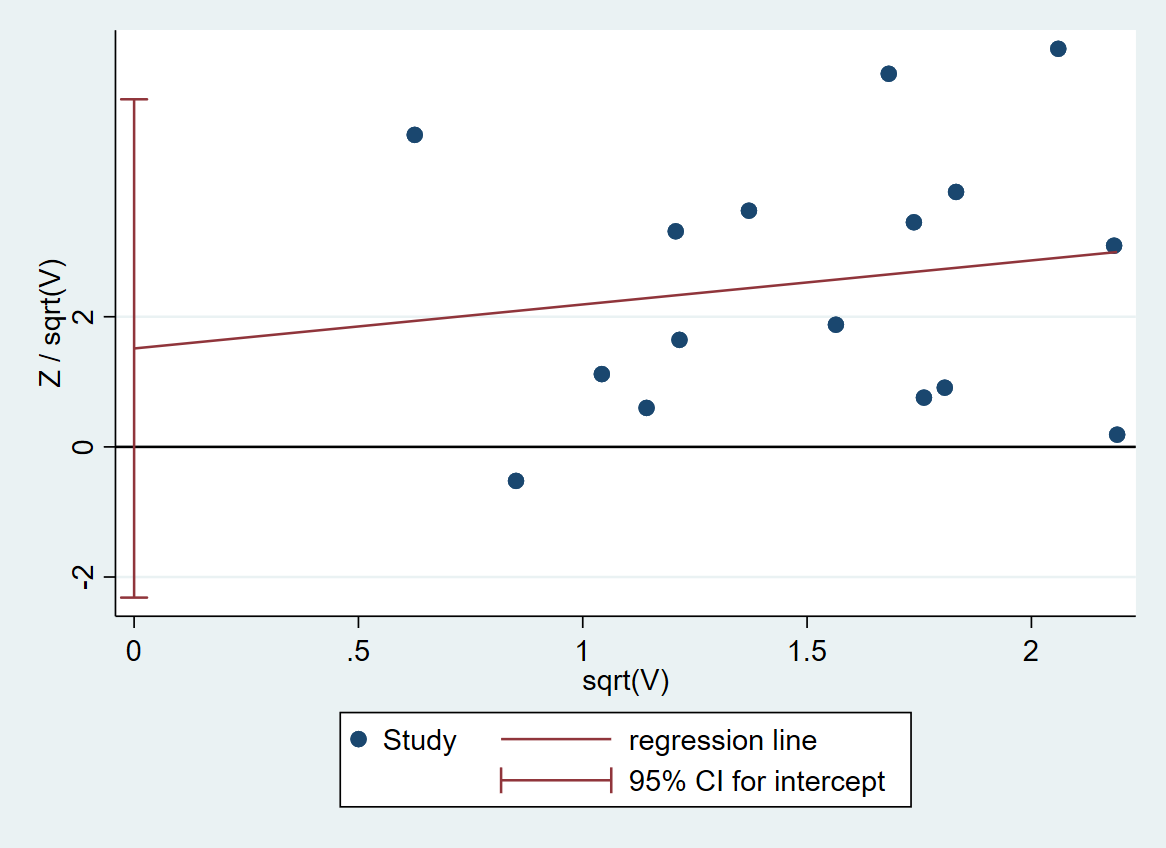


**Supplementary figure 6.** The Harbord test for ctDNA prediction of locally advanced rectal cancer recurrence.


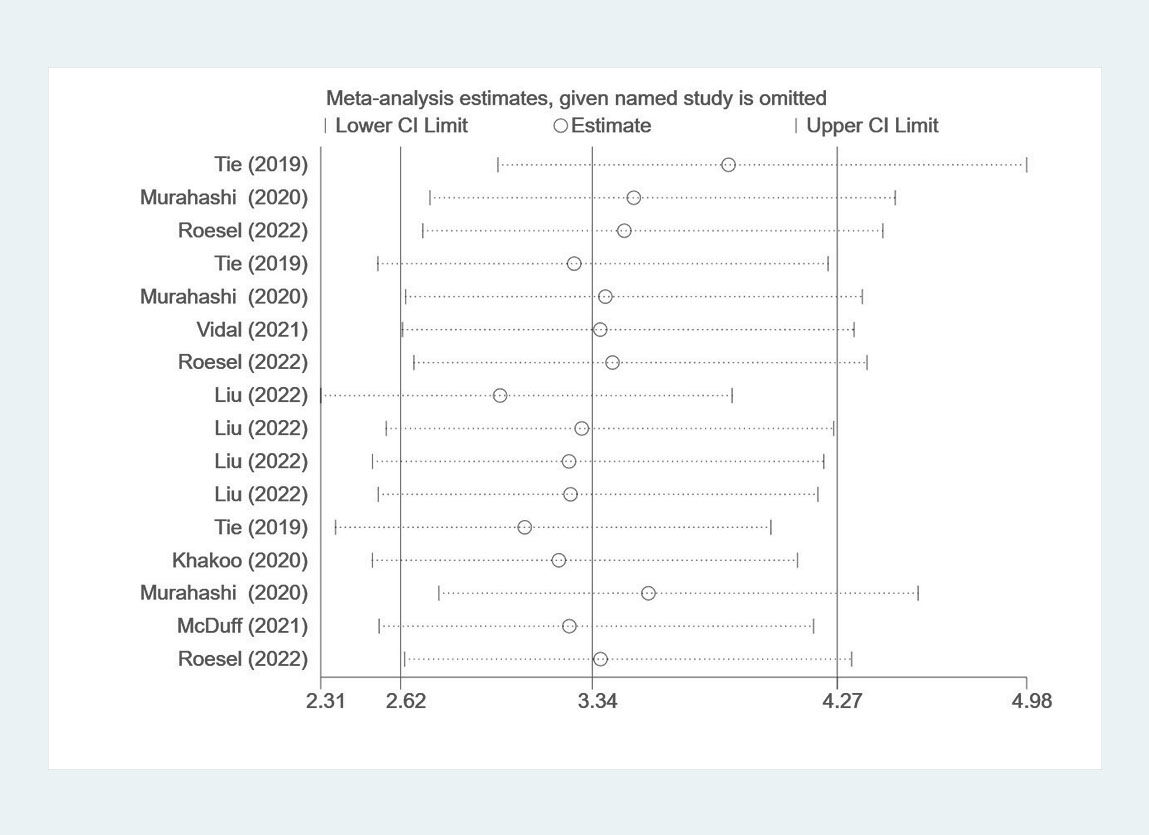


**Supplementary figure 7.** The sensitivity analysis for ctDNA prediction of locally advanced rectal cancer recurrence.


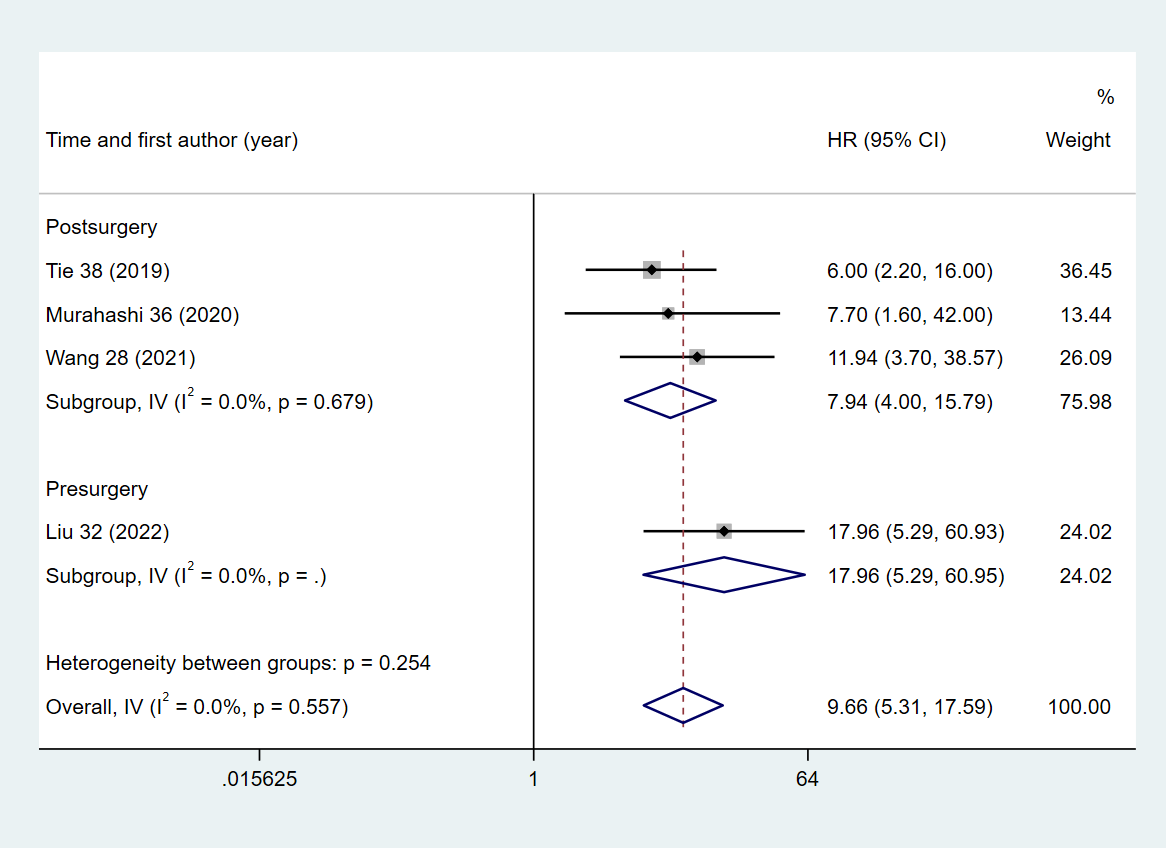


**Supplementary figure 8.** Forest plot of multivariate progression-free survival.


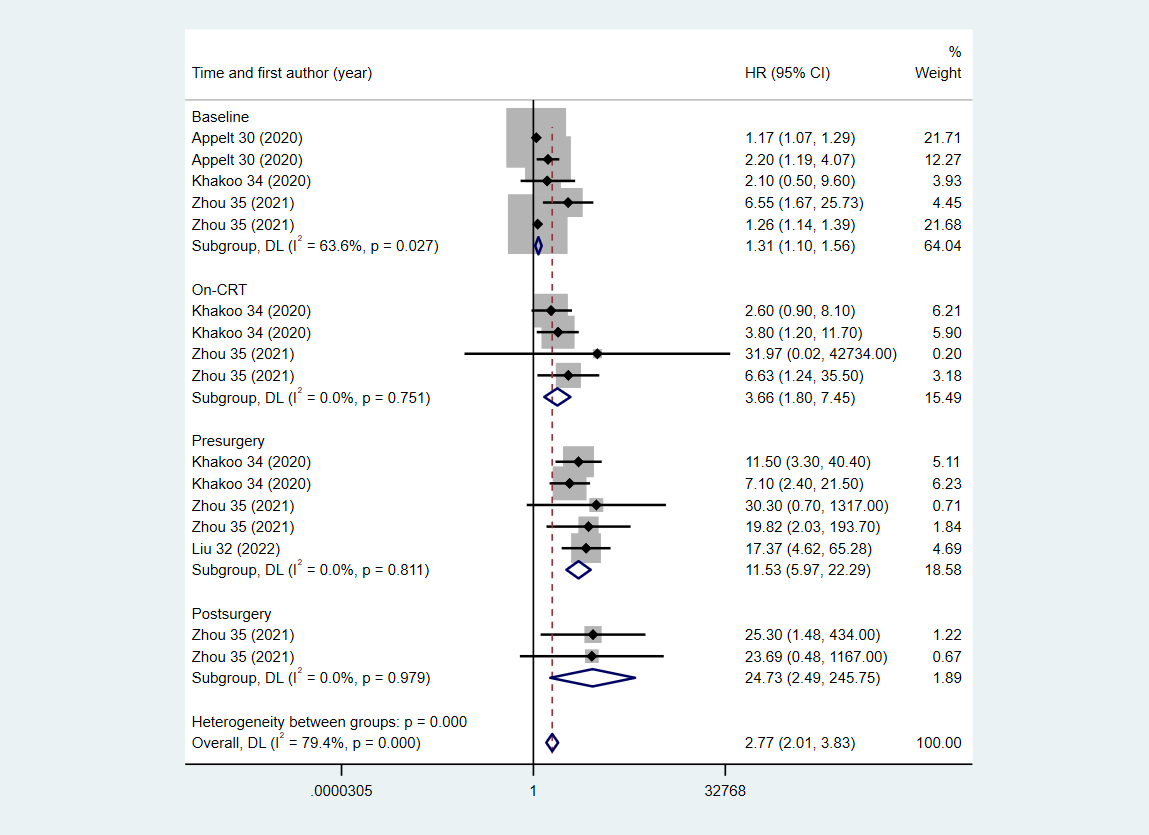


**Supplementary figure 9.** Forest plot on ctDNA prediction of metastases-free survival with a pooled effect hazard ratio.

**Supplementary table 1.** Definition of the clinical outcomes and treatment strategy in the studies included in the systematic review and meta-analysis

| Author | Year | ctDNA testing time | Definition of pCR | Long-term endpoint definition | Treatment strategy |
| --- | --- | --- | --- | --- | --- |
| Sclafani | 2018 | Baseline | CR was defined using the RECIST v1.1 criteria. | PFS was measured from date of randomisation to date of first progression/relapse or death from any cause. OS was measured from date of randomisation to death from any cause. | Neoadjuvant chemotherapy with CapeOx followed by chemoradiotherapy total mesorectal excision and adjuvant CapeOx with or without cetuximab. |
| Tie | 2019 | Baseline  Presurgery  Postsurgery | pCR was defined as ypT0N0. | RFS was measured from date of surgery to documented first recurrence or death as a result of colorectal cancer. | Preoperative long-course fluoropyrimidine-based chemoradiotherapy will be followed by TME surgery. The use of chemotherapy after surgery was at the discretion of the treating clinician. |
| Appelt | 2020 | Baseline | Complete response was defined as TRG1 | For OS, death from any cause was considered an event. For MFS, the first incidence of distant metastasis was counted as an event,irrespective of any previous locoregional recurrence. | Patients received 50.4 Gy in 28 fractions with concomitant oral UFT and L-leucovorin, plus an additional 2×5 Gy brachytherapy tumor boost in the experimental arm. TME was performed after the end of CRT, and adjuvant chemotherapy was delivered. |
| Khakoo | 2020 | Baseline  On-CRT  Presurgery  Postsurgery | CR was defined according to RECIST version1.1. | MFS was measured from study entry to development of metastases or death from any cause. DFS was measured from date of surgery until relapse or death from any cause. OS was measured from study entry to death from any cause or censored by last follow-up if alive. | CRT consisted of capecitabine alongside 50.4-54 Gy radiotherapy. In the absence of disease progression with metastatic disease, treatment options included: surgery, organ preservation, or neoadjuvant chemotherapy. |
| Murahashi | 2020 | Baseline  Presurgery  Postsurgery | pCR was defined as ypT0N0M0. | NA | Thirty-three patients received standard CRT. Nine patients received SRT. Four patients with upper rectal cancer received NAC. Twenty-three patients with lower rectal cancer received FOLFOX+Bmab followed by CRT. 16 patients with cT3N1M0 received a combination of SRT, followed CapeOx. |
| Pazdirek | 2020 | Baseline | TRG according to Dworak (TRG 3, near CR; TRG 4, CR) | NA | All patients underwent NCRT consisting of 50.4 Gy of radiation and concomitant administration of capecitabine. |
| Vidal | 2021 | Baseline  presurgery | The pCR rate was defined as ypT0ypN0 | DFS was defined as the time from randomization until recurrence, second primary tumor, or death, whichever occurred first, independently of whether patient underwent surgery or not. OS was defined as the time from randomization to death from any cause. | Patients (induction chemotherapy with aflibercept plus mFOLFOX6 or induction chemotherapy with mFOLFOX6 alone) were administered, followed by CRT with capecitabine. TME surgery was performed after CRT completion. |
| Zhou | 2021 | Baseline  On-CRT  Presurgery  Postsurgery | ypTRG was based on the College of American Pathologists grading system. | MFS was calculated from the date of nCRT started until distant metastasis occurred. | All the participants received the long-term neoadjuvant radiotherapy with three cycles of nCT. The enrolled patients were randomly assigned to receive capecitabine or CapeOx during nCRT. |
| Wang | 2021 | Baseline  On-CRT  Presurgery  Postsurgery | pCR was defined as pathological T0N0M0 and pTRG = 0 | NA | Patients received nCRT (50Gy/25 fractions; concurrent capecitabine + irinotecan) and 1 cycle of CAPIRI, followed by TME and 5 cycles of CapeOx. |
| McDuff | 2021 | Baseline  Presurgery  Postsurgery | NA | PFS was estimated from the date of surgery until the time of radiographic progression or date of last follow-up. | Patients were treated with long-course chemoradiation consisting of 45 Gy to the pelvis followed by a boost to the mesorectum to 50.4 Gy in 1.8 Gy fractions with concurrent capecitabine or infusional fluorouracil. |
| Roesel | 2022 | Baseline  On-CRT  Presurgery  Postsurgery | NA | NA | Patients received long course 5-fluorouracil based chemotherapy plus 50.4 Gy radiotherapy and TME surgery. |
| Liu | 2022 | Baseline  On-CRT  Presurgery | NA | RFS was defined as the time from the date of randomization to the first occurrence of localregional failure or distant metastasis. | Patients enrolled were randomly assigned in a 1:1 ratio to short-course preoperative radiotherapy with NAC and preoperative long-course chemoradiotherapy. |

**Abbreviation**: CR, complete response; RECIST, response evaluation criteria in solid tumours; pCR, pathological complete response; DFS, disease-free survival; NA, not available; OS, overall survival; UFT, tegafluorouracil; CRT, chemo-radiotherapy; NAC, neoadjuvant chemotherapy; PFS, progression-free survival; RFS, recurrence-free survival; CapeOx, oxaliplatin and capecitabine; TME, total mesorectal excision; TRG, tumor regression grade; MFS, metastasis-free survival; mrTRG, MRI tumor regression grade; LRFS, local recurrence-free survival; SRT, short-course radiotherapy; FOLFOX, fluorouracil+leucovorin+oxaliplatin; CAPIRI, capecitabine+irinotecan.

**Supplementary table 2.** Newcastle-Ottawa Scale for Quality Assessment of the eligible studies

| Author | Year | Selection | Comparability | Outcome | Total |
| --- | --- | --- | --- | --- | --- |
| Sclafani | 2018 | 2 | 2 | 2 | 6 |
| Tie | 2019 | 4 | 2 | 2 | 8 |
| Appelt | 2020 | 3 | 2 | 3 | 8 |
| Khakoo | 2020 | 2 | 2 | 2 | 6 |
| Murahashi | 2020 | 3 | 2 | 1 | 6 |
| Pazdirek | 2020 | 2 | 2 | 1 | 5 |
| Vidal | 2021 | 3 | 2 | 2 | 7 |
| Zhou | 2021 | 4 | 2 | 2 | 8 |
| Wang | 2021 | 4 | 2 | 2 | 8 |
| McDuff | 2021 | 2 | 2 | 2 | 6 |
| Roesel | 2022 | 2 | 2 | 2 | 6 |
| Liu | 2022 | 3 | 2 | 2 | 7 |

**Search details**

Two authors independently conducted a comprehensive and systematic search of PubMed, Embase, and Web of Science, respectively, up to September 6, 2022.

**PubMed**

#1: Circulating Tumor DNA 8266

#2: ctDNA 10,295

#3: (Circulating Tumor) AND (DNA) 11,065

#4: (Tumor DNA) AND (Circulating) 11,065

#5: Cell Free Tumor DNA 20,045

#6: (Cell-Free Tumor) AND (DNA) 5,474

#7: (Tumor DNA) AND (Cell-Free) 5,474

#8: #1 or #2 or #3 or #4 or #5 or #6 or #7

((((((Circulating Tumor DNA) OR (ctDNA)) OR ((Circulating Tumor) AND (DNA))) OR ((Tumor DNA) AND (Circulating))) OR (Cell Free Tumor DNA)) OR ((Cell-Free Tumor) AND (DNA))) OR ((Tumor DNA) AND (Cell-Free)) 25,616

#9: Rectal cancer 78,778

#10: Rectal Neoplasms 71,031

#11: Locally advanced rectal cancer 6,630

#12: #9 or #10 or #11

((Locally advanced rectal cancer) OR (Rectal cancer)) OR (Rectal Neoplasms) 78,778

#13: Recurrence risk 180,645

#14: Minimal residual disease 29,735

#15: Prognosis 2,132,406

#16: Neoadjuvant chemoradiotherapy 44,740

#17: Chemoradiotherapy 35,284

#18: Pathological complete response 60,405

#19: # 13 or #14 or #15 or #16 or #17 or #18

(((((Pathological complete response) OR (Chemoradiotherapy)) OR (Neoadjuvant chemoradiotherapy)) OR (Prognosis)) OR (Minimal residual disease)) OR (Recurrence risk) 2,303,951

#20: #8 and #12 and #19

((((((((Circulating Tumor DNA) OR (ctDNA)) OR ((Circulating Tumor) AND (DNA))) OR ((Tumor DNA) AND (Circulating))) OR (Cell Free Tumor DNA)) OR ((Cell-Free Tumor) AND (DNA))) OR ((Tumor DNA) AND (Cell-Free))) AND (((Locally advanced rectal cancer) OR (Rectal cancer)) OR (Rectal Neoplasms))) AND ((((((Pathological complete response) OR (Chemoradiotherapy)) OR (Neoadjuvant chemoradiotherapy)) OR (Prognosis)) OR (Minimal residual disease)) OR (Recurrence risk)) 115

**Embase**

#1: circulating AND tumor AND dna 18,345

#2: ctdna 8,932

#3: circulating AND tumor AND dna 18,345

#4: 'cell free' AND tumor AND dna 7,295

#5: cell AND free AND tumor AND dna 27,656

#6: #1 or #2 or #3 or #4 or #5 41,505

#7: rectal AND cancer 87,669

#8: rectal AND neoplasms 5,302

#9: locally AND advanced AND rectal AND cancer 8,992

#10: #7 or #8 or #9 88,518

#11: recurrence AND risk 260,518

#12: minimal AND residual AND disease 43,325

#13: prognosis 1,149,033

#14: neoadjuvant AND chemoradiotherapy 17,945

#15: chemoradiotherapy 83,848

#16: pathological AND complete AND response 14,764

#17: #11 or #12 or #13 or #14 or #15 or #16 1,446,239

#18: #6 and #10 and #17 244

**Web of science**

#1: Circulating Tumor (Topic) and DNA (Topic) 14,985

#2: Tumor DNA (Topic) and Circulating (Topic) 14,985

#3: Cell Free Tumor DNA (Topic) 31,550

#4: Cell-Free Tumor (Topic) and DNA (Topic) 6,901

#5: Tumor DNA (Topic) and Cell-Free (Topic) 6,901

#6: Circulating Tumor DNA (Topic) 14,985

#7: ctDNA (Topic) 5,662

#8: #1 or #2 or #3 or #4 or #5 or #6 or #7 43,305

#9: Rectal cancer (Topic) or Rectal Neoplasms (Topic) or Locally advanced rectal cancer (Topic) 73,958

#10: Recurrence risk (Topic) or Minimal residual disease (Topic) or Prognosis (Topic) or Neoadjuvant chemoradiotherapy (Topic) or Chemoradiotherapy (Topic) or Pathological complete response (Topic) 1,038,138

#11: #8 and #8 and #10 190
